# Targeting RPLP2 Triggers DLBCL Ferroptosis by Decreasing FXN Expression

**DOI:** 10.3390/biomedicines13061320

**Published:** 2025-05-28

**Authors:** Jiaxing Guo, Bokang Yan, Lingshu Li, Yuanhao Peng, Weiwei Lai, Chanjuan Shen

**Affiliations:** 1 Department of Hematology, Zhuzhou Hospital Affiliated to Xiangya School of Medicine, Central South University, Zhuzhou 412007, China; 2 Laboratory of Hematology, Department of National Clinical Key Specialty, Zhuzhou Hospital Affiliated to Xiangya School of Medicine, Central South University, Zhuzhou 412007, China; 3 Department of Pathology, Zhuzhou Hospital Affiliated to Xiangya School of Medicine, Central South University, Zhuzhou 412007, China; 4 NHC Key Laboratory of Carcinogenesis, Central South University, Changsha 410078, China; 5 Hunan Provincial Key Laboratory of Pediatric Orthopedics, The Affiliated Children’s Hospital of Xiangya School of Medicine, Central South University, Changsha 410007, China; 6 College of Medicine, Jishou University, Jishou 416106, China

**Keywords:** DLBCL, RPLP2, FXN, ferroptosis, Destruxin b, doxorubicin

## Abstract

**Background/Objectives:** Ribosomal Protein Lateral Stalk Subunit P2 (RPLP2), an important ribosomal protein, is mainly involved in modulating protein synthesis and plays an essential role in the carcinogenesis of many cancers. However, its precise impact on diffuse large B-cell lymphoma (DLBCL) remains unknown. **Methods:** This study utilized siRNA to knock down RPLP2, aiming to investigate its role in DLBCL progression. RT-qPCR and immunohistochemistry (IHC) were employed to assess RPLP2 and frataxin (FXN) expression levels in DLBCL. CCK8 and colony formation assays measured cell proliferation inhibition upon RPLP2 deletion, while transwell migration assays analyzed reduced cell motility. Lipid ROS and iron assays quantified ferroptosis markers to elucidate RPLP2’s regulation of FXN-mediated ferroptosis. Xenograft mouse models validated tumor suppression effects in vivo. **Results:** Here, we reveal that elevated RPLP2 expression is significantly correlated to unfavorable prognosis in DLBCL patients. In addition, we demonstrate that RPLP2 deletion dramatically reduces the cell proliferation and migration of DLBCL. Besides, knockdown of RPLP2 triggers ferroptosis via regulating ferroptosis suppressor FXN activity. Moreover, we discover that Destruxin b could target RPLP2 to suppress the development of DLBCL. Lastly, the combination of Destruxin b with Dox remarkably improves the anti-tumor effect. **Conclusions:** In general, the present study reveals the oncogenic role of RPLP2 in DLBCL, uncovers an unrecognized regulatory axis of ferroptosis, and identifies a specific inhibitor targeting RPLP2 to restrain DLBCL progression, suggesting that RPLP2 could be a potential target for DLBCL treatment.

## 1. Introduction

Diffuse large B-cell lymphoma (DLBCL), characterized by exceptionally rapid disease progression and aggressive clinical behavior, is the most common subtype of non-Hodgkin’s lymphoma (NHL), accounting for more than one-third of all NHL cases worldwide [1,2]. Despite the emergence of first-line chemical immunotherapy, R-CHOP, including doxorubicin (Dox), rituximab, cyclophosphamide, vincristine, and prednisone, approximately 40% of cases progress to refractory or relapsed DLBCL [3,4]. Therefore, identifying novel effective therapeutic targets is critical to guide clinical practice.

Ferroptosis, a novel type of regulated cell death in terms of morphology, genetics, and biochemistry, is characterized by iron dependent lipid peroxidation and reactive oxygen species (ROS) accumulation [5,6]. Studies indicate that the high susceptibility of cancer cells to ferroptosis contributes to overcoming resistance to existing therapy alternatives [7]. Research increasingly shows that the regulation of ferroptosis-related proteins, such as FSP1, GPX4, and MT1G, plays an essential role in the progression of DLBCL [8,9,10]. However, whether other key protein may regulate ferroptosis to affect the tumorigenesis of DLBCL remains largely unknown.

Ribosomal Protein Lateral Stalk Subunit P2 (RPLP2), a component of the human ribosome P complex, plays an important role in accelerating protein biosynthesis via recruiting translation factors [11,12]. Mounting evidence proves that RPLP2 is upregulated and correlated with poor outcomes in many cancers, such as gynecological tumors, lung cancer, and liver cancer [13,14,15]. Additionally, Luo et al. previously reported its preliminary clinical associations in DLBCL [16]. However, the specific role of RPLP2 in the development of DLBCL is unknown, and the possibility of targeting RPLP2 with specific inhibitors for cancer treatment remains to be explored. Gene set enrichment analysis (GSEA) from a previous study shows that RPLP2 has significant effects on the ferroptosis-related pathway “Oxidative Phosphorylation” in pediatric acute myeloid leukemia [17]. Additionally, it has been reported that the deletion of RPLP2 could lead to the abnormal accumulation of ROS in gynecological tumors [13], and promotes ferroptosis in HCC cells [18]. However, whether RPLP2 is essential for the regulation of ferroptosis in DLBCL remains elusive.

Frataxin (FXN), a key regulator of ferroptosis, is closely related to the biogenesis of iron–sulfur clusters (ISCs) in mitochondria, and plays a key role in modulating iron homeostasis [19]. As for cancers, knockdown of FXN could activate the iron starvation response and make breast cancer cells more sensitive to ferroptosis [20]. In addition, inhibiting FXN expression significantly enhances ferroptosis by accelerating free iron accumulation and lipid peroxidation [21], whereas FXN itself inhibits ethanol-induced ferroptosis in HCC cells through maintaining mitochondrial iron homeostasis and redox balance [22]. While the RPLP2–FXN–ferroptosis axis is known in solid tumors, its role in lymphoid malignancies (particularly DLBCL) remains unexplored, and its validation in DLBCL represents an important gap.

Therefore, this inspires us to explore the carcinogenic effects of RPLP2 in DLBCL, investigate the specific role of the RPLP2-FXN axis in the regulation of ferroptosis in DLBCL for the first time, and further illustrate the possibility of targeting RPLP2 to improve therapeutic efficacy in DLBCL.

## 2. Materials and Methods

### 2.1. Cell Culture

DLBCL cell lines SU-DHL8 and OCI-LY3 were purchased from Bluef (Shanghai, China) Biotechnology Development Co., Ltd. DLBCL cell lines OCI-LY10 and U2932, along with a normal B cell line (NBC), were provided by the Cancer Research Institute of Central South University. The cells were cultured in DMEM medium (Gibco, Grand Island, NY, USA) supplemented with 1% Penicillin-Streptomycin-Amphotericin B and 10% fetal bovine serum (FBS) at 37 °C in a 5% CO_2_ atmosphere.

### 2.2. RNA Inference, Plasmids and Chemicals

The specific sequences of small interfering RNAs targeting RPLP2 were as follows: RPLP2-siRNA1: 5′-GGUUAUUAGUGAGCUGAAUTT-3′; RPLP2-siRNA2: 5′-GGAGUCUGAA GAGUCAGACTT-3′. FXN cDNA was amplified by PCR from 293T cells and verified by sequencing. Lipofectamine^®^ 2000 (Invitrogen, Carlsbad, CA, USA) was applied for siRNA and plasmid transfections in accordance with the manufacturer’s recommendations.

The cells were exposed to 10 μM of the Erastin (571203-78-6, MedChemExpress, Monmouth Junction, NJ, USA) for 24 h. The cells were treated with 5 nM (SU-DHL8) or 10 nM (OCI-LY3) of apoptosis inducer VCR (S1241, Selleck) for 48 h. The cells were incubated with 50 nM of autophagy inducer Rapamycin (S1039, Selleck, Houston, TX, USA) for 48 h. The cells were primed with 100 ng/mL pyroptosis inducer LPS (S7850, Selleck) for 6 h, followed by 10 μM of Nigericin (S6653, Selleck) for 2 h. Ferroptosis inhibitor Ferrostatin-1 (Fer-1) (S7243) (10 μM) was purchased from Selleck. Dox (D1515) was supplied by Sigma-Aldrich. Destruxin b (DB) was obtained according to the experiment methods described previously [23].

### 2.3. RT-qPCR

TRIzol (Takara, Kusatsu, Japan) was applied for isolating total RNA, and PrimeScriptTM RT reagent kit (Takara, Kusatsu, Japan) containing gDNA Eraser was utilized to synthesize cDNA. The ABI 7500 and the FastStart Universal SYBR Green Master were used to perform RT-qPCR. In order to normalize relative gene expression levels, β-actin was employed. The sequences of RT-qPCR primers are listed in Table S1.

### 2.4. Immunohistochemistry (IHC)

The detailed protocol has been described previously [24]. The primary antibodies used for IHC in the present study included RPLP2 (1:100, 823061, Zenbio, Chengdu, China), FXN (1:100, DF6590, Affinity, Liyang, China) and 4-HNE (1:100, Abcam, ab46545, Cambridge, UK). DLBCL patient tissues were collected from Zhuzhou Central Hospital from 1 January 2017 to 30 June 2024. Bokang Yan, a pathological technician at the Department of Pathology of Zhuzhou Central Hospital, verified the DLBCL biopsies.

### 2.5. Cell Proliferation and Migration Assays

The cell proliferation analysis was performed using a CCK8 assay and clone formation assay. For the CCK8 assay, 1000 cells were seeded into each well of 96-well plates, following by the addition of 10 μL CCK8 solution to each well and incubation at 37 °C for 2 h. Then, a microplate reader was used to measure the absorbance at 450 nm. For the clone formation assay, 3000 cells were mixed with methylcellulose (RnD)-conditioned DMEM with 10% FBS. After approximately two weeks, colonies were methanol fixed and stained with 0.5% crystal violet. All experiments were performed in triplicate.

For the migration analysis, 3 × 10^6^ cells were starved in serum-free DMEM medium and added to the upper chamber, and the lower chamber were added with 600 μL complete medium. Then, the plate was incubated for 8 h at 37 °C, and the number of migrated cells was detected by the CCK8 assay.

### 2.6. Measurement of Lipid ROS and Iron Assays

For the lipid ROS assay, the complete protocols have been introduced previously [18]. The lipid ROS measurement was conducted by treating cells with 10 µM C11-BODIPY (D3861, Thermo Fisher, Waltham, MA, USA).

The detailed protocols for measurement of ferrous iron (Fe^2+^) and total iron have been described in another manuscript [25]. The experiment was performed using an Iron Assay Kit (Sigma-Aldrich, St. Louis, MO, USA).

### 2.7. Nude Mice and Study Approval

The animal experiments were performed under the approved protocols of the Experimental Animal Welfare and Ethics Committee of Central South University. The female nude mice aged 4 weeks were provided by Hunan SJA Laboratory Animal Co., Ltd. 4 × 10^6^ SU-DHL8 cells were injected to the back of each mouse, and tumor sizes were measured every 3 days until the end of experiment. The nude mice were placed in a sterile, pathogen free, temperature and humidity-controlled environment with daily health monitoring by researchers certified in laboratory animal care. On day 25 post-injection, following the first observation of significant tumor growth disparities among experimental groups, all mice were humanely euthanized via carbon dioxide asphyxiation in accordance with institutional animal welfare protocols.

### 2.8. Statistical Analyses

The data were presented in the form of the mean ± SD or SEM. Statistical analysis was performed using unpaired, two-tailed *t*-tests, the Wilcoxon rank sum test, Cox regression, GSEA, and Spearman’s correlation analysis or two-way analysis of variance (ANOVA). All cell-based experiments included 3 biological replicates, each with 3 technical replicates. GraphPad Prism 8.0 and Microsoft Excel were applied for all statistical analyses. A *p*-value < 0.05 was regarded as statistically significant.

## 3. Results

### 3.1. RPLP2 Is Upregulated and Correlated with Poor Prognosis in DLBCL

**Figure 1:**
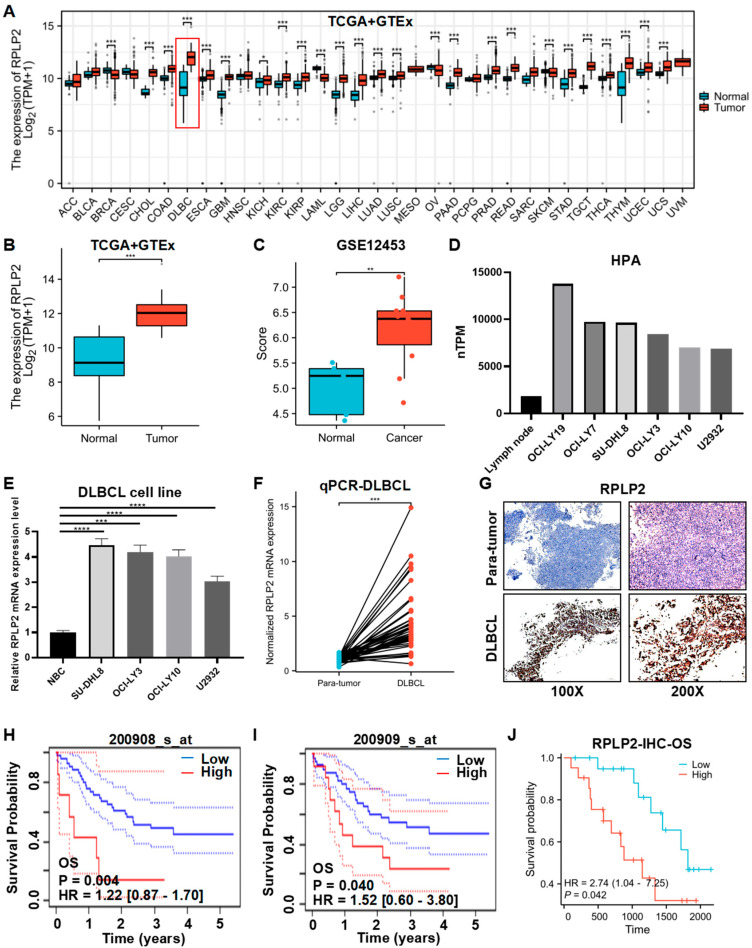
RPLP2 is upregulated and correlated with poor outcomes in DLBCL. (**A**) RPLP2 mRNA expression levels in pan-cancer cases from the TCGA and GTEx databases. (**B**,**C**) The mRNA levels in RPLP2 in normal lymphoid tissues and DLBCL tissues from the TCGA and GTEx databases (**B**) or the GSE12453 database (**C**). (**D**) The RPLP2 mRNA levels in lymph node and DLBCL cell lines from the HPA database. (**E**) RT-qPCR for detecting the RPLP2 mRNA levels in a normal B cell line and DLBCL cell lines. (**F**) RT-qPCR for detecting the RPLP2 mRNA levels in the ZCH DLBCL cohort (n = 84), including adjacent tissues (n = 42) and DLBCL tissues (n = 42). (**G**) IHC analysis of RPLP2 expression in the ZCH DLBCL cohort. (**H**–**J**) Kaplan–Meier curves for patients’ OS in DLBCL, classified by RPLP2 mRNA levels obtained from the E-TABM-346 dataset (**H**,**I**) or the ZCH DLBCL cohort (**J**). Data are presented as mean values ± SEM (**A**–**C**) or SD (**E**). N = 3 independent repeats (**E**,**F**). Statistical analysis was performed using the Wilcoxon rank sum test (**A**–**C**), unpaired, two-tailed *t*-tests (**E**,**F**), or Cox regression (**H**–**J**). * *p* < 0.05, ** *p* < 0.01, *** *p* < 0.001, **** *p* < 0.0001.

We first conducted a pan-cancer analysis of RPLP2 mRNA expression based on TCGA and GTEx databases, and the results indicated that RPLP2 significantly increased in 21 different types of cancer, especially in DLBCL (Figure 1A,B). And the analysis results of the GSE12453 dataset also showed that RPLP2 was upregulated in DLBCL (Figure 1C). Additionally, Human Protein Atlas (HPA) data showed that RPLP2 mRNA expression was higher in DLBCL cell lines compared to lymph nodes (Figure 1D), and the staining intensity of RPLP2 was greater in non-Hodgkin lymphoma (NHL) than in lymph node tissue (Figure S1A). Then, we performed an RT-qPCR experiment to further prove that RPLP2 exhibited higher expression levels in DLBCL cell lines (Figure 1E) and tissues (Figure 1F). Moreover, the IHC test also demonstrated that RPLP2 had higher expression level in DLBCL tissues compared to para-tumor tissues (Figure 1G and Figure S1B). Lastly, we utilized a K-M plotter based on the E-TABM-346 dataset (Figure 1H,I) and Zhuzhou Central Hospital’s (ZCH) DLBCL cohort (Figure 1J) to show that DLBCL patients with increased RPLP2 expression had worse outcomes regarding overall survival (OS). And the receiver operating curve (ROC) indicated that RPLP2 had great performance (AUC = 0.964) in distinguishing DLBCL from normal controls (Figure S1C). Taken together, these findings proved that the high expression levels of RPLP2 in DLBCL are closely associated with unfavorable outcomes.

### 3.2. RPLP2 Deletion Suppresses DLBCL Progression

**Figure 2:**
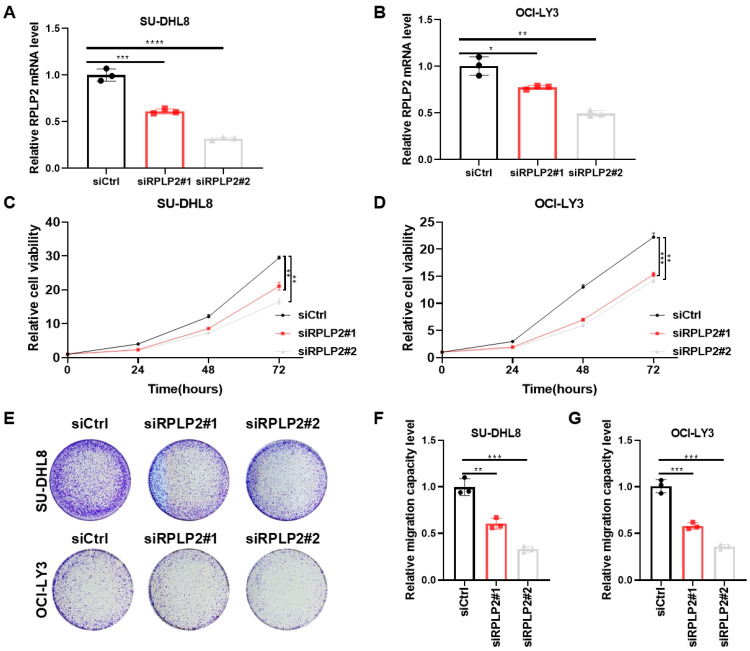
RPLP2 deletion suppresses DLBCL growth. (**A**,**B**) RT-qPCR for detecting siRPLP2#1 and siRPLP2#2 transfection efficiency in SU-DHL8 (**A**) and OCI-LY3 cells (**B**). (**C**,**D**) CCK8 assay for identifying the effect of RPLP2 knockdown on the proliferation of SU-DHL8 (**C**) and OCI-LY3 (**D**) cells. (**E**) The colony formation assay was applied for assessing the impact of RPLP2 deletion on the clone formation capability of DLBCL cells. (**F**,**G**) A Transwell migration assay of SU-DHL8 (**F**) and OCI-LY3 (**G**) cells with RPLP2 silencing. Data are presented as mean values ± SD, n = 3 independent repeats (**A**–**D**,**F**,**G**). Statistical analysis was performed using unpaired, two-tailed *t*-tests (**A**,**B**,**F**,**G**) or two-way ANOVA (**C**,**D**). * *p* < 0.05, ** *p* < 0.01, *** *p* < 0.001, **** *p* < 0.0001.

To further explore the biological functions of RPLP2 in DLBCL, we deleted RPLP2 in SU-DHL8 and OCI-LY3 cells (Figure 2A,B). CCK8 analysis showed that RPLP2 knockdown dramatically inhibited the proliferation of SU-DHL8 (Figure 2C) and OCI-LY3 cells (Figure 2D). Colony formation assay showed that RPLP2 silencing decreased the colony formation ability of DLBCL cells (Figure 2E). A Transwell migration assay demonstrated that RPLP2 deletion significantly reduced the migration of SU-DHL8 (Figure 2F) and OCI-LY3 cells (Figure 2G). Conclusively, these results illustrated that RPLP2 deletion suppressed DLBCL progression significantly.

### 3.3. RPLP2 Deletion Promotes Ferroptosis of DLBCL Cells

**Figure 3:**
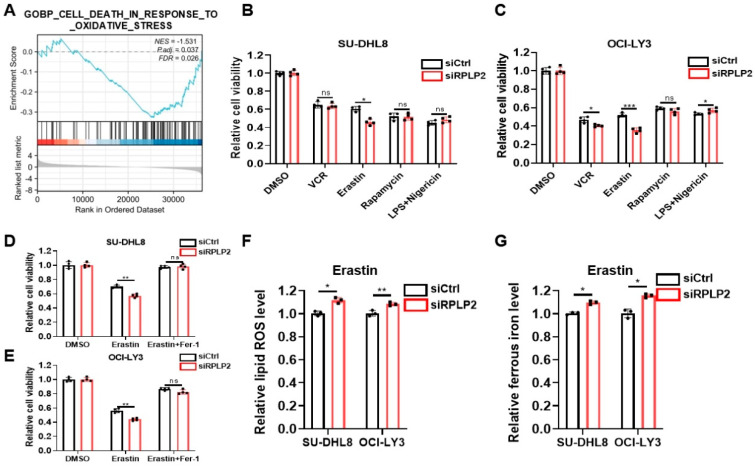
RPLP2 knockdown triggers ferroptosis. (**A**) The GSEA analysis of TCGA data enriched the “Cell death in response to oxidative stress” pathway. (**B**,**C**) The cell viability of SU-DHL8 (**B**) and OCI-LY3 (**C**) cells with RPLP2 deletion after the addition of apoptosis inducer VCR, ferroptosis inducer Erastin, autophagy inducer Rapamycin, and pyroptosis inducer LPS + Nigericin was assessed using a CCK8 assay. (**D**,**E**) CCK8 assays for the detection of the response of SU-DHL8 (**D**) and OCI-LY3; (**E**) RPLP2-knockdown cells treated with Erastin and Fer-1. (**F**,**G**) Levels of lipid ROS (**F**) and ferrous iron (**G**) in the RPLP2 knockdown DLBCL cells. Data are presented as mean values ± SD, n = 3 independent repeats (**B**–**G**). Statistical analysis was performed using GSEA (**A**) or unpaired, two-tailed *t*-tests (**B**–**G**). ns nonsignificant *p* > 0.05, * *p* < 0.05, ** *p* < 0.01, *** *p* < 0.001.

The deletion of RPLP2 has been shown to induce autophagy in gynecological tumors and promote the ferroptosis of HCC cells [13,18]. Thus, it was of great interest to explore whether RPLP2 played an important role in regulating the cell fate of DLBCL. We first performed GSEA to reveal that genes in the RPLP2 low-expression group were more involved in the “Cell death in response to oxidative stress” pathway (Figure 3A). Then, we compared the efficacy of several inducers of major types of oxidative stress-related cell death, including apoptosis inducer VCR, ferroptosis inducer Erastin, autophagy inducer Rapamycin, and pyroptosis inducer LPS + Nigericin. The results proved that ferroptosis inducer Erastin exhibited the most remarkable cell-death-inducing effect, comparing to other inducers (Figure 3B,C). Furthermore, we treated RPLP2-knockdown SU-DHL8 and OCI-LY3 cells with Erastin. The results of the CCK8 analysis (Figure 3D,E) demonstrated that RPLP2 deletion triggered the ferroptosis of DLBCL cells. Additionally, we found that RPLP2 knockdown significantly increased the intracelluar lipid ROS (Figure 3F) and ferrous iron (Figure 3G). In conclusion, our results demonstrated that RPLP2 deletion promoted the ferroptosis of DLBCL cells.

### 3.4. RPLP2 Regulates Ferroptosis by Increasing FXN Activity

**Figure 4:**
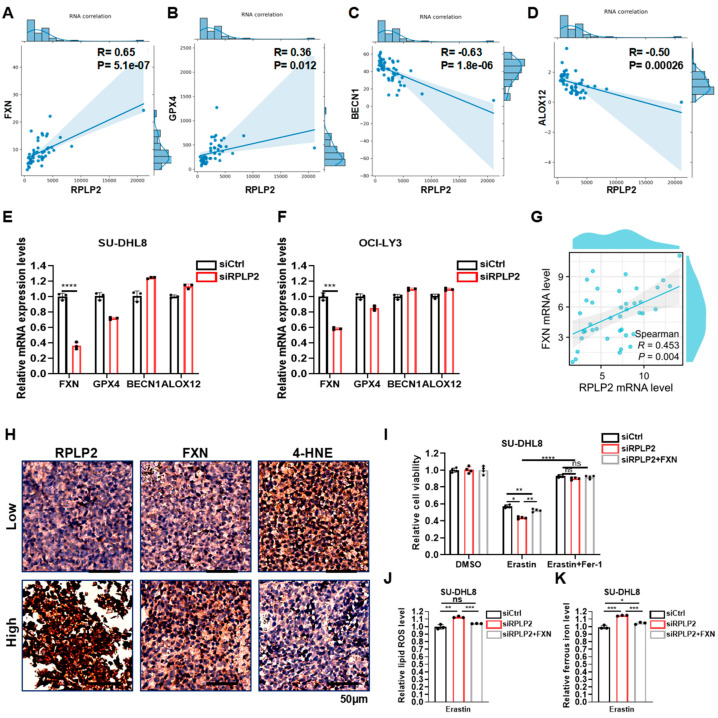
RPLP2 regulates ferroptosis via enhancing FXN expression. (**A**–**D**) Correlation analyses of RPLP2 expression and key ferroptosis regulators FXN (**A**), GPX4 (**B**), BECN1 (**C**), and ALOX12 (**D**) in DLBCL based on FerrDb. (**E**,**F**) The relative mRNA expression levels of FXN, GPX4, BECN1, and ALOX12 in SU-DHL8 (**E**) and OCI-LY3 cells (**F**) with RPLP2 knockdown. (**G**) Spearman’s correlation analysis for analyzing the relationship between RPLP2 and FXN in the ZCH DLBCL cohort. (**H**) An IHC test on 12 DLBCL tissues to identify the correlation between RPLP2 and FXN or 4-HNE. (**I**) The response of RPLP2-knockdown of SU-DHL8 cells with FXN overexpression to Erastin and Fer-1 was presented using a CCK8 assay. (**J**,**K**) Lipid ROS (**J**) and ferrous iron (**K**) levels in RPLP2-deleted SU-DHL8 cells with FXN overexpression were detected. Data are presented as mean values ± SD, n = 3 independent repeats (**E**,**F**,**I**–**K**). Statistical analysis was performed using Spearman’s correlation analysis (**A**–**D**,**G**) or unpaired, two-tailed *t*-tests (**E**,**F**,**I**–**K**). ns nonsignificant *p* > 0.05, * *p* < 0.05, ** *p* < 0.01, *** *p* < 0.001, **** *p* < 0.0001.

To investigate the concrete molecular mechanism of RPLP2 in regulating ferroptosis, we conducted a correlation analysis of RPLP2 and key ferroptosis regulators in TCGA DLBCL samples to show that RPLP2 is significantly positively associated with the ferroptosis suppressor genes FXN and GPX4 (Figure 4A,B and Figure S2A) and negatively linked to ferroptosis-driver genes BECN1 and ALOX12 (Figure 4C,D and Figure S2B). Then, we further found the significant reduction in FXN in SU-DHL8 (Figure 4E) and OCI-LY3 cells (Figure 4F) with the absence of RPLP2. Additionally, Spearman’s correlation analysis between RPLP2 and FXN in the ZCH DLBCL cohort also indicated that RPLP2 positively correlated with FXN (Figure 4G). Moreover, the IHC experiment further proved that RPLP2 protein levels are positively linked to FXN and negatively correlated with the ferroptosis biomarker 4-HNE (Figure 4H and Figure S2C). Lastly, we utilized rescue experiments to determine whether RPLP2 regulated ferroptosis by enhancing FXN activity. By forced expression of FXN into RPLP2 knockdown cells via plasmid transfection, the CCK8 assay indicated that FXN overexpression markedly rescued cells from RPLP2-dependent ferroptosis (Figure 4I and Figure S2D). Similarly, elevated intracellular lipid ROS (Figure 4J and Figure S2E) and ferrous iron (Figure 4K and Figure S2F) could be reversed by RPLP2 ectopic expression. Together, our findings suggest that RPLP2 inhibited the ferroptosis of DLBCL cells by enhancing FXN expression.

### 3.5. Destruxin b Targets RPLP2 to Suppress Tumor Progression via Triggering Ferroptosis

**Figure 5:**
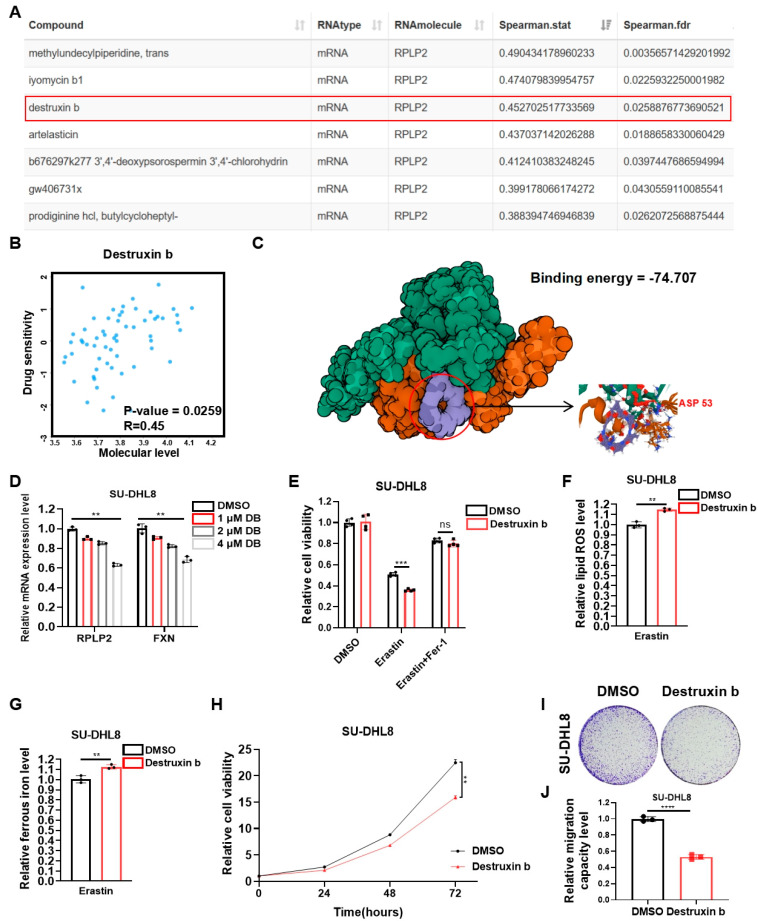
Destruxin b targets RPLP2 to suppress DLBCL progression via promoting ferroptosis. (**A**) Correlation analysis between drug sensitivity and RPLP2 mRNA expression based on RNAactDrug database. (**B**) Correlation between Destruxin b drug sensitivity and RPLP2 mRNA expression was analyzed using RNAactDrug database. (**C**) Potential binding model of Destruxin b and RPLP2 was predicted using Dockeasy web tool. (**D**) RT-qPCR was used to detect RPLP2 and FXN levels in SU-DHL8 cells treated with increased concentration of Destruxin b. (**E**) CCK8 assay for analyzing response of SU-DHL8 cells treated with Destruxin b to Erastin and Fer-1. (**F**,**G**) Levels of lipid ROS (**F**) and ferrous iron (**G**) were detected in SU-DHL8 cells treated with Destruxin b. (**H**–**J**) CCK8 assay (**H**), colony formation assay, and (**I**) and Transwell assay (**J**) of SU-DHL8 cells treated with Destruxin b. Data are presented as mean values ± SD, n = 3 independent repeats (**D**–**H**,**J**). Statistical analysis was performed using Spearman’s correlation analysis (**A**,**B**), unpaired, two-tailed *t*-tests (**D**–**G**,**J**), or two-way ANOVA (**H**). ns nonsignificant *p* > 0.05, ** *p* < 0.01, *** *p* < 0.001, **** *p* < 0.0001.

The essential role of RPLP2 in regulating DLBCL progression prompted us to further explore potential highly sensitive chemicals that specifically target RPLP2. First, we performed correlation analysis between the drug sensitivity and mRNA expression of RPLP2. The results indicated that the drug sensitivity of chemicals, including Methylundecylpiperidine, trans, Iyomycin b1, Destruxin b, and Artelasticin, increased with the elevation of RPLP2 mRNA expression (Figure 5A,B). Considering the inhibitory effect of Destruxin b on a variety of cancers, including NHL [26,27,28,29], we further utilized the molecular protein interaction prediction model to predict the possibility of binding Destruxin b to RPLP2. The binding model exhibited extremely low binding energy at “74.707 kcal/mol”, indicating a high possibility of binding between them (Figure 5C). Then, we used an increasing concentration of Destruxin b to treat SU-DHL8 cells to prove that Destruxin b suppresses RPLP2 expression, which leads to a reduction in FXN (Figure 5D). Furthermore, the results demonstrated that Destruxin b could target RPLP2 to induce ferroptotic cell death (Figure 5E) and increase lipid ROS (Figure 5F) and ferrous ion (Figure 5G). And the biological functional experiments suggested that the treatment of Destruxin b inhibited the cell proliferation (Figure 5H), colony formation (Figure 5I), and migration (Figure 5J) of SU-DHL8 cells. In summary, we found that Destruxin b degraded RPLP2 to suppress DLBCL progression by promoting ferroptosis.

### 3.6. Destruxin b Improves the Anti-Tumor Effect of DOX

**Figure 6:**
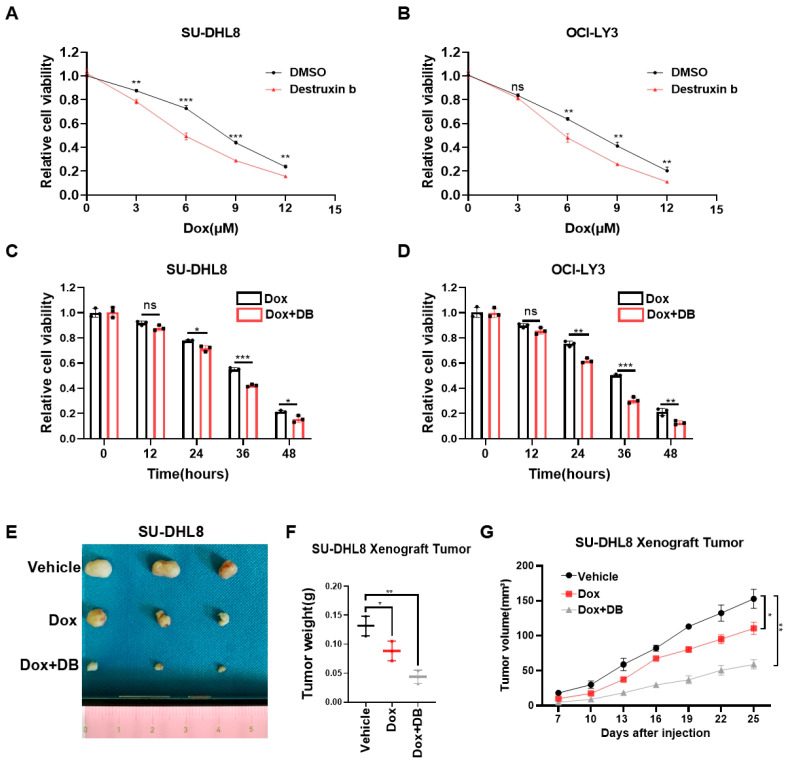
Co-treatment of Destruxin b with doxorubicin further improves anti-tumor effects. (**A**,**B**) Cell viability of SU-DHL8 (**A**) and OCI-LY3 (**B**) cells exposed to increased concentrations of Dox, with or without Destruxin b, for 24 h was assessed by CCK8 assay. (**C**,**D**) Cell viability of SU-DHL8 (**C**) and OCI-LY3 (**D**) cells exposed to Dox (3 μM), with or without Destruxin b, for indicated time was assessed by CCK8 assay. (**E**–**G**) Parental SU-DHL8 cells were transplanted on nude mice treated with vehicle control, DOX alone, or in combination with Destruxin b intraperitoneally (10 mg/kg, 3 times/week) (n = 3 mice per group) (**E**,**F**). Tumor sizes were measured every three days until mice were euthanized (**G**). Data are presented as mean values ± SD (**A**–**D**) or SEM (**G**). N = 3 independent repeats (**A**–**D**,**F**,**G**). Statistical analysis was performed using unpaired, two-tailed *t*-tests (**A**–**D**,**F**) or two-way ANOVA (**G**). ns nonsignificant *p* > 0.05, * *p* < 0.05, ** *p* < 0.01, *** *p* < 0.001.

Dox, the first-line chemotherapy drug for DLBCL, had serious side effects due to the high doses required [4]. Thus, we further evaluated whether Destruxin b could act synergistically with Dox to have an additive therapeutic effect for DLBCL. At the beginning, we treated SU-DHL8 and OCI-LY3 cells with increased concentrations of Dox in combination with or without Destruxin b for 24 h. The CCK8 assays showed that the combination of Dox and Destruxin b significantly improved the inhibitory effect on cell viability (Figure 6A,B). Then, we added Dox, with or without Destruxin b, to SU-DHL8 and OCI-LY3 cells for 12 h, 24 h, 36 h, and 48 h, respectively. Consistently, the cell viability decreased more in the combination group (Figure 6C,D). Lastly, we used the xenograft nude mice model composed of SU-DHL8 cells to demonstrate that administration of Destruxin b in combination with Dox remarkably inhibited tumor growth in vivo compared to Dox alone (Figure 6E–G). In short, the combination of Destruxin b with Dox exhibited an inspiring additive anticancer effect.

## 4. Discussion

DLBCL, a highly heterogeneous group of B-cell lymphomas in molecular pathology and cytogenetics, is the most common pathological subtype of NHL globally [30]. Despite recent advances in radiotherapy, chemotherapy (R-CHOP regimen), and immune checkpoint inhibitors (ICIs), the prognosis of DLBCL patients still remains unfavorable [31,32,33]. Thus, it is important to explore new potential therapeutic targets for the treatment of DLBCL. RPLP2 contributes to protein synthesis as an integral part of the ribosomal stalk [34] and has been proven to play an essential role in regulating the occurrence and progression of the multiple cancers such as breast cancer, lung cancer, and liver cancer [14,15,35]. However, the concrete role of RPLP2 in DLBCL has not been investigated so far. Here, we first detected the expression level and prognostic significance of RPLP2 in DLBCL. Then, we investigated the effect of RPLP2 on DLBCL progression and elucidated the regulatory mechanism of RPLP2 in ferroptosis. Lastly, the potential of targeting RPLP2 to improve therapeutic efficacy in DLBCL was explored.

As a novel candidate oncogene, RPLP2 has been demonstrated to be upregulated and closely linked to the tumorigenesis of many cancers [18,36,37,38]. For example, previous studies indicate that RPLP2 deficiency leads to stress-induced autophagy of gynecological tumors [13], and RPLP2 could facilitate HCC tumor growth by regulating glycolysis through the activation of the PI3K/AKT/HIF-1α pathway [15]. However, the specific role of RPLP2 in the progression of DLBCL remains elusive. Here, we initially analyzed the expression level of RPLP2 in DLBCL using the TCGA + GTEx, GEO, and HPA databases to discover that RPLP2 was significantly overexpressed in DLBCL tissues. Then, we further conducted RT-qPCR and IHC experiments to prove that RPLP2 expression was elevated in DLBCL compared with adjacent tissues. Moreover, a K-M plotter based on the E-TABM-346 dataset or ZCH DLBCL cohort was utilized to indicate that DLBCL patients with elevated RPLP2 expression had a worse prognosis of OS. Lastly, we used in vitro biological functional experiments to demonstrate that RPLP2 deletion dramatically inhibited the growth and migration of DLBCL. Together, RPLP2 is overexpressed and exhibits a remarkable pro-tumor effect in DLBCL.

Ferroptosis, a novel type of iron-dependent programmed cell death marked by unrestricted lipid peroxidation, is considered to be an effective target for cancer treatment [7,39]. FXN has been demonstrated to be a key regulator of ferroptosis through regulating iron homeostasis and mitochondrial function in several cancers [20,21]. Recent studies indicate that RPLP2 deletion significantly correlates with ROS accumulation in gynecological tumors and induces ferroptosis mediated by GPX4 in HCC [13,18]. However, the relationship between RPLP2 and ferroptosis in DLBCL and related molecular mechanisms has not been studied. In the present research, we revealed that RPLP2 knockdown triggered the ferroptosis of DLBCL cells using CCK8 analysis. Additionally, we found that the deletion of RPLP2 increased intracellular lipid ROS and ferrous iron. Moreover, the results of correlation analysis, RT-qPCR, and IHC tests showed that RPLP2 positively correlated with pivotal ferroptosis suppressor FXN in DLBCL. The rescue experiment further proved that RPLP2 regulated the ferroptosis of DLBCL cells through increasing FXN expression. Conclusively, this is the first study to elucidate the significant role of the RPLP2-FXN axis in modulating ferroptosis in DLBCL.

The critical role of RPLP2 in DLBCL progression propels us to further explore the possibility of targeting RPLP2, specifically with highly sensitive chemicals, to improve the prognosis of DLBCL patients. We first used the RNAactDrug database and prediction model of molecular–protein interaction to screen Destruxin b (a cyclic peptide with anticancer activity identified from the entomopathogenic fungus, Metarhizium anisopliae [40]), which showed significant drug sensitivity and extremely low binding energy with RPLP2. Then, we proved that both RPLP2 and FXN decreased with increasing Destruxin b concentration. Additionally, treating SU-DHL8 cells with Destruxin b led to ferroptotic cell death as well as elevated lipid ROS and ferrous iron. Furthermore, Destruxin b suppressed the proliferation, migration, and clone formation of DLBCL cells. In summary, Destruxin b could inhibit DLBCL growth by inducing ferroptosis via the RPLP2-FXN axis.

Dox, the main cytotoxic component of R-CHOP, is an anti-tumor chemotherapy agent which inhibits topoisomerase II, thus interfering with DNA replication [41]. However, high doses of Dox are needed to be effective due to its short half-life and low bioavailability, and high doses are often associated with severe side effects, especially cardiotoxicity [42,43]. This study indicated that the combination of Destruxin b with Dox showed an exciting additive anti-tumor effect, which may be assumed to be linked to Destruxin b sensitizing DLBCL cells by triggering ferroptosis, thus making them more easily affected by Dox’s cytotoxic effects. But the concrete molecular mechanism of this additive effect remains unexplored.

**Figure 7:**
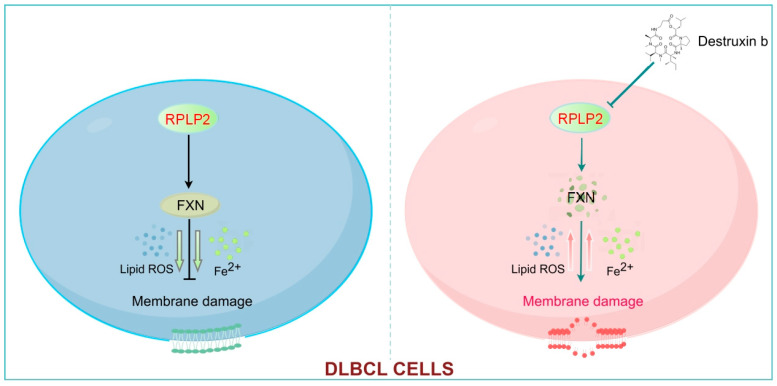
RPLP2 inhibits the ferroptosis of DLBCL cells by enhancing FXN activity. RPLP2 protects DLBCL from ferroptosis by increasing expression of FXN. Targeting RPLP2 by Destruxin b could be a promising strategy for DLBCL therapy.

In general, this study proved that increased an expression level of RPLP2 is an adverse prognostic factor in DLBCL. In addition, we revealed that RPLP2 plays a pro-tumor role in the progression of DLBCL. Furthermore, the RPLP2-FXN axis is demonstrated to exhibit significant impacts on modulating ferroptosis. Moreover, our results showed that Destruxin b could suppress DLBCL tumor growth by targeting RPLP2, and the co-treatment of Destruxin b with Dox further improves the anti-tumor effect of Dox (Figure 7). Therefore, RPLP2 could be a promising therapeutic target for DLBCL. However, the specific regulatory mechanism of RPLP2 on FXN has not been discussed in this study, which needs to be further illustrated in our next research.

## Data Availability

Correspondence and requests for materials or data used and analyzed in the present study should be addressed to Chanjuan Shen or Bokang Yan.

## Supplementary Materials

The following supporting information can be downloaded at: https://www.mdpi.com/article/10.3390/biomedicines13061320/s1.
