# Supplementary material for: Targeting RPLP2 Triggers DLBCL Ferroptosis by Decreasing FXN Expression
Source: Biomedicines. 2025 May 28;13(6):1320. doi: 10.3390/biomedicines13061320 (PMC12189634; doi:10.3390/biomedicines13061320)
Supplement: Supplementary file 1 [file biomedicines-13-01320-s001.zip › biomedicines-3611781-supplementary.pdf]

Supplementary Materials for

Targeting RPLP2 triggers DLBCL ferroptosis by decreasing FXN expression

This file includes:

Figures S1 to S2

Table S1

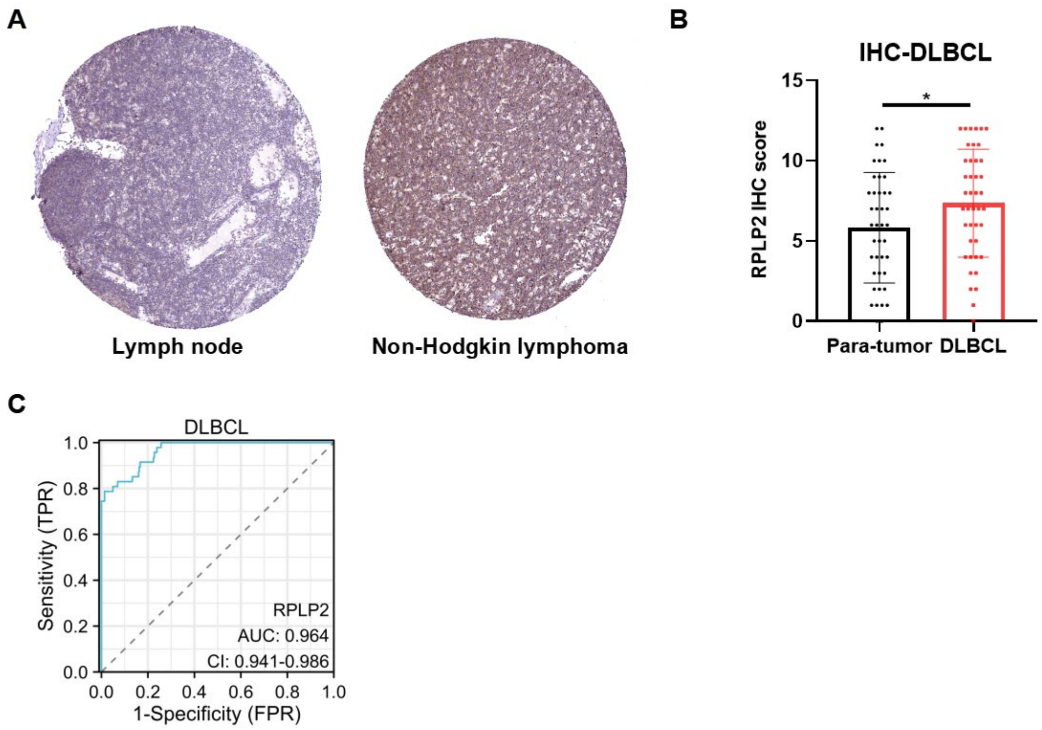

Figure S1. RPLP2 is overexpressed in DLBCL and has great diagnostic accuracy. (A) The different expression of RPLP2 between lymph node and non-Hodgkin lymphoma based on HPA database. (B) IHC scores presenting RPLP2 levels in para-tumor tissues and DLBCL tissues. (C) ROC analysis of RPLP2 in DLBCL. Data are presented as mean values ± SD. Statistical analysis was performed using unpaired, two-tailed t-test (B). *$P < 0.05$.

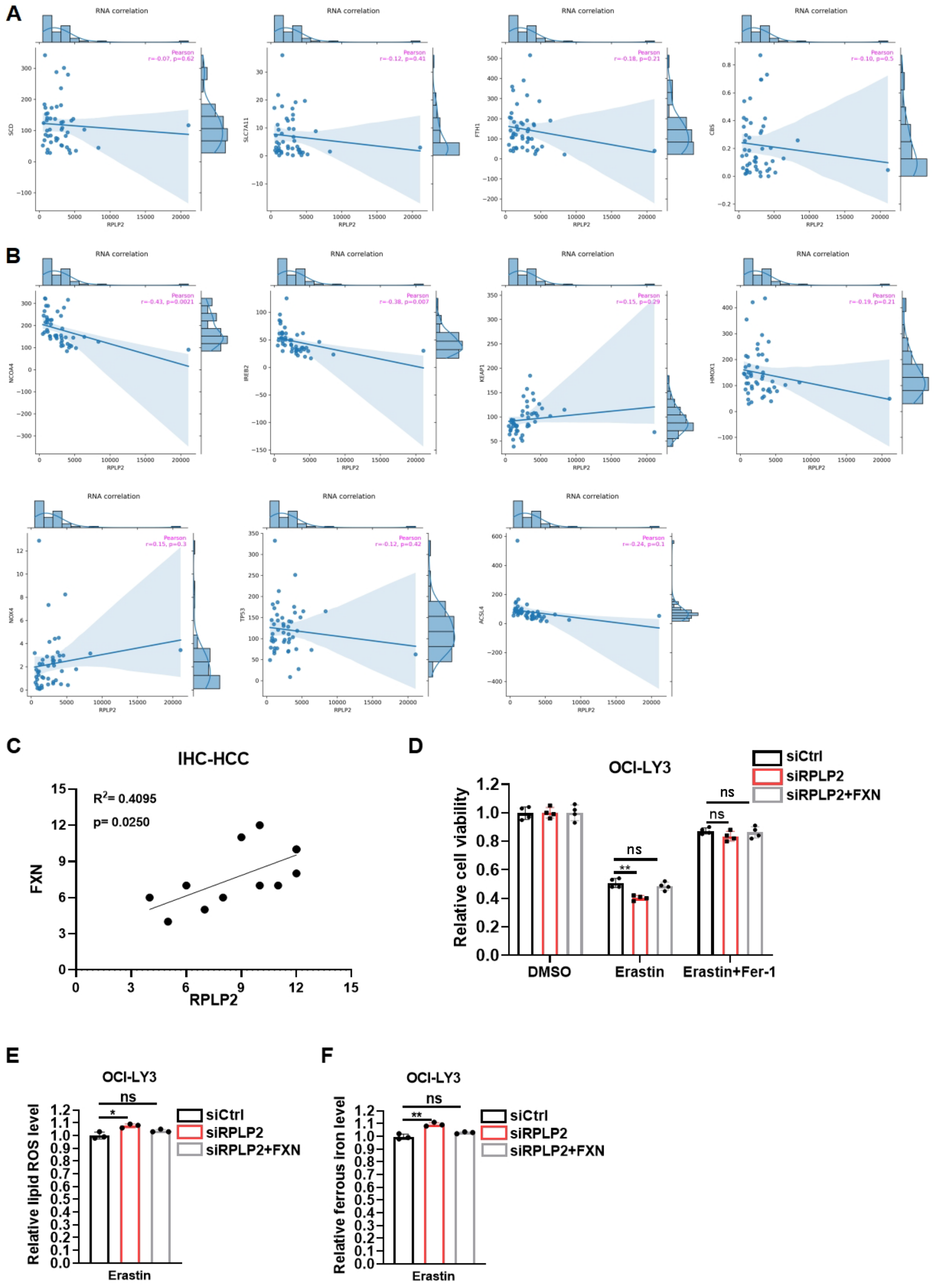

Figure S2. RPLP2 inhibits ferroptosis of OCI-LY3 cells via enhancing FXN expression. (A, B) Correlation analysis between RPLP2 and key suppressors (A) or drivers (B) of ferroptosis. (C) Correlation analysis of the protein levels of

RPLP2 and FXN was performed in 12 DLBCL tissues. (D) The response of OCI-LY3 cells knockdown of RPLP2 with FXN overexpression to RSL-3 and Fer-1 was presented by CCK8 assay. (E, F) Lipid ROS (E) and ferrous iron (F) levels in RPLP2 deletion OCI-LY3 cells with FXN overexpression were detected. Data are presented as mean values ± SD, n = 3 independent repeats (D-F). Statistical analysis was performed using Spearman (A-C) or unpaired, two-tailed t-test (D-F). ns nonsignificant $P > 0.05$, $^{*}P < 0.05$, $^{**}P < 0.01$.

Table S1. The sequences of the primers for RT-qPCR used in the present study.

Name	Forward premier (5’-3’)	Reverse premier (5’-3’)
RPLP2	TCTTGGACAGCGTGGGTATCGA	CAGCAGGTACACTGGCAAGCTT
FXN	GCCTCAACCAGATTTGGAATGTC	AGTCCAGCGTTTCCTCTGCTAG
GPX4	ACAAGAACGGCTGCGTGGTGAA	GCCACACACTTGTGGAGCTAGA
BECN1	CTGGACACTCAGCTCAACGTCA	CTCTAGTGCCAGCTCCTTTAGC
NCOA4	GCTTGCTATTGGTGGAGTTCTCC	GCCATACCTCACGGCTTCTAAG
β-actin	CACCATTGGCAATGAGCGGTTC	AGGTCTTTGCGGATGTCCACGT
